# Hypertriglyceridemia, Metabolic Syndrome, and Cardiovascular Disease in HIV-Infected Patients: Effects of Antiretroviral Therapy and Adipose Tissue Distribution

**DOI:** 10.1155/2012/201027

**Published:** 2011-08-22

**Authors:** Jeroen P. H. van Wijk, Manuel Castro Cabezas

**Affiliations:** ^1^Department of Internal Medicine, University Medical Center, P.O. Box 85500, 3508 GA Utrecht, The Netherlands; ^2^Department of Internal Medicine, Center for Diabetes and Vascular Medicine, St. Franciscus Gasthuis Rotterdam, P.O. Box 10900, 3004 BA Rotterdam, The Netherlands

## Abstract

The use of combination antiretroviral therapy (CART) in HIV-infected patients has resulted in a dramatic decline in AIDS-related mortality. However, mortality due to non-AIDS conditions, particularly cardiovascular disease (CVD) seems to increase in this population. CART has been associated with several metabolic risk factors, including insulin resistance, low HDL-cholesterol, hypertriglyceridemia and postprandial hyperlipidemia. In addition, HIV itself, as well as specific antiretroviral agents, may further increase cardiovascular risk by interfering with endothelial function. As the HIV population is aging, CVD may become an increasingly growing health problem in the future. Therefore, early diagnosis and treatment of cardiovascular risk factors is warranted in this population. This paper reviews the contribution of both, HIV infection and CART, to insulin resistance, postprandial hyperlipidemia and cardiovascular risk in HIV-infected patients. Strategies to reduce cardiovascular risk are also discussed.

## 1. Introduction

The widespread use of combination antiretroviral therapy (CART) has led to a dramatic and sustained reduction in the morbidity and mortality associated with HIV infection and has transformed this disease into a chronic condition [1, 2]. CART generally consists of two nucleoside analogue reverse-transcriptase inhibitors (NRTIs) and a protease inhibitor (PI) or nonnucleoside analogue reverse-transcriptase inhibitor (NNRTI). Despite an enormous decrease in AIDS-related mortality, CART has been associated with changes in body fat distribution and several metabolic risk factors, such as hypertriglyceridemia, low HDL-cholesterol, and insulin resistance [3–5]. Moreover, recent studies have shown that prolonged use of CART is associated with an increased risk of cardiovascular disease (CVD) [6, 7]. As treatment of HIV infection has become more successful, CVD may become an increasingly growing health problem in HIV-infected patients. This review focuses on the underlying mechanisms and characteristics of dyslipidemia, insulin resistance, and CVD in HIV-infected patients. 

## 2. Lipodystrophy

CART in HIV-infected patients is strongly associated with changes in body fat distribution, often referred to as lipodystrophy [3–5]. Lipodystrophy is characterized by subcutaneous fat loss, visceral fat accumulation, and development of a buffalo hump. Subcutaneous fat loss is most noticeable in the face, limbs, and buttocks and may occur independently of visceral fat accumulation. The prevalence of lipodystrophy varies widely, from 10 to 80 percent, and is mainly dependent on the type and duration of CART and the criteria used for diagnosing lipodystrophy [3–5]. Severe forms of lipodystrophy, especially lipoatrophy, can be disfiguring and stigmatizing and often lead to suboptimal adherence to CART. All classes of antiretroviral agents may be related to the development of lipodystrophy, but the prevalence and severity of lipodystrophy are increased mostly in patients treated with the combination of NRTIs and a PI [3–5]. The etiology of lipodystrophy appears to be multifactorial, including HIV drug inhibitory effects on adipocyte differentiation and alteration of mitochondrial functions. PIs impede adipocyte differentiation through altered expression and nuclear localization of sterol regulatory element-binding protein-1 (SREBP-1) and peroxisome proliferator-activated receptor-*γ* (PPAR-*γ*), which are essential for adipogenesis [8]. NRTIs may induce mitochondrial dysfunction and apoptosis of adipocytes by inhibition of mitochondrial DNA polymerase-*γ* and depletion of mitochondrial DNA [9].

## 3. Dyslipidemia

The natural course of HIV infection is characterized by reductions in HDL-cholesterol and LDL-cholesterol and an increase in triglycerides (TGs) [10]. Elevated TGs are due to a combination of hepatic very low-density lipoprotein (VLDL) overproduction and reduced TG clearance [10, 11]. Hypertriglyceridemia is related to poor virological control and increased levels of TNF-*α* [10, 11]. TNF-*α* interferes with free fatty acid (FFA) metabolism and lipid oxidation and attenuates insulin-mediated suppression of lipolysis [11]. The nutritional state of HIV-infected patients, including weight loss and protein depletion, contributes to reduced HDL-cholesterol and LDL-cholesterol levels [10, 11]. 

Following the introduction of CART, more pronounced atherogenic changes in the lipid profile, including increases in TG and LDL-cholesterol, and a decrease in HDL-cholesterol, have been observed [3–5]. In addition, increases in apolipoprotein B (apoB) have been found, often associated with the predominance of atherogenic small dense LDL [3–5]. In a large cross-sectional study, the prevalence of hypercholesterolemia (>6.2 mmol/L), hypertriglyceridemia (>2.3 mmol/L), and low HDL-cholesterol (<0.9 mmol/L) was 10 to 27 percent, 23 to 40 percent, and 19 to 27 percent, respectively, depending on the antiretroviral regimen [12]. The pathogenesis of CART-related dyslipidemia is complex and involves various drug-induced effects, in association with hormonal and immunological influences. Especially PI therapy has been associated with dyslipidemia. The most pronounced changes in the lipid profile have been observed with intensive booster doses of ritonavir [13]. Amprenavir and nelfinavir have intermediate effects on plasma lipids, while indinavir, saquinavir, and lopinavir have minor effects on plasma lipids [3–5, 13]. Atazanavir does not negatively affect the lipid profile [14]. The effects of PIs on the lipid profile seem to be drug-related, because an interaction with the host response to HIV or changes in body composition has been excluded by several short-term studies conducted in HIV-negative volunteers [15–17]. In these subjects, ritonavir increased the concentrations of plasma TG, apoB, and VLDL-cholesterol as early as 2 weeks after administration [15]. Administration of lopinavir/ritonavir to healthy HIV-negative volunteers for 4 weeks resulted in increased TG and decreased HDL-cholesterol levels [16]. In contrast, Noor et al. showed that administration of indinavir for 4 weeks to HIV-negative subjects did not result in significant changes in lipoproteins, TG, or FFA levels but caused insulin resistance independent of increases in visceral adipose tissue [17]. Apparently, changes in the lipid profile develop as early as weeks after administration of a PI, independent of HIV infection or body fat distribution, suggesting direct effects of PIs on lipid metabolism. 

The mechanism of PI-induced dyslipidemia is not fully understood but is probably multifactorial. First, it has been suggested that PIs suppress the breakdown of the nuclear form of SREBP-1 in the liver [18]. SREBP-1 is a master transcriptional regulator and regulates the expression of genes involved in FFA, TG, and cholesterol biosynthesis. Hence, hepatic accumulation of SREBP-1 could result in increased hepatic de novo lipogenesis. For example, ritonavir has been shown to increase the level of active ADD-1/SREBP-1 protein during adipogenesis [18]. Similarly, indinavir and nelfinavir, but not amprenavir, altered adipose cell differentiation and SREBP-1 nuclear localization in an adipose cell line [19]. Second, PIs seem to suppress proteasomal breakdown of nascent apoB in the liver, leading to increased VLDL secretion [20]. In human hepatoma (HepG2) cells, treatment with therapeutically relevant concentrations of ritonavir or saquinavir protected nascent apoB from intracellular degradation [20]. Whether this is also the case for the other PIs is not known. A third proposed pathway is based upon the structural similarity between the catalytic region of HIV-1 protease and two homologous proteins involved in lipid metabolism: cytoplasmic retinoic acid-binding protein type 1 (CRABP-1) and low density lipoprotein-receptor-related protein (LRP) [4]. CRABP-1 is involved in the conversion of retinoic acid to cis-9-retinoic acid, which binds the retinoid X receptor- (RXR-)PPAR-*γ* heterodimer, stimulating adipocyte differentiation and proliferation. PIs are thought to bind to CRABP-1 and inhibit the formation of cis-9-retinoic acid, leading to reduced PPAR-*γ* activity and peripheral lipoatrophy [4]. Impaired FFA storage capacity in adipose tissue and increased flux of circulating lipids may upregulate hepatic VLDL production and, hence, contribute to hyperlipidemia. LRP normally binds to lipoprotein lipase (LPL) on capillary endothelium, which hydrolyses FFA from TG, promoting their accumulation in adipocytes. Remnants of TG-rich lipoproteins (TRLs) are removed from the circulation by the LDL-receptor and the hypothetical remnant receptor in the liver. Binding of PIs to LRP would impair hepatic remnant uptake and TG clearance by the endothelial LRP-LPL complex [4]. Evidence supporting this concept has been provided in apoE3-Leiden transgenic mice treated with ritonavir, demonstrating decreased LPL-mediated TG clearance as well as impaired uptake of TG-derived FFA in adipose tissue, which may contribute to lipodystrophy [21].

PI-sparing CART may also affect the lipid profile, although cholesterol and TG levels generally rise less in comparison with regimens containing a PI [12, 22]. Of the NNRTIs, efavirenz is associated with higher levels of cholesterol and TG than is nevirapine, whereas both increase HDL-cholesterol [23]. The severity and prevalence of dyslipidemia in HIV-infected patients may also depend on HIV disease stage and the concomitant presence of lipodystrophy and insulin resistance [3–5, 12]. The presence of lipodystrophy in HIV-infected patients has been associated with accelerated lipolysis, hepatic reesterification, and hypertriglyceridemia [24]. However, although it is likely that increased FFA release from adipose tissue contributes to the increase in hepatic VLDL synthesis, other factors must be involved, because insulin-induced suppression of lipolysis and systemic FFA availability did not normalize the VLDL-TG secretion rate in a kinetic study [25].

## 4. Postprandial Lipemia

**Figure 1:**
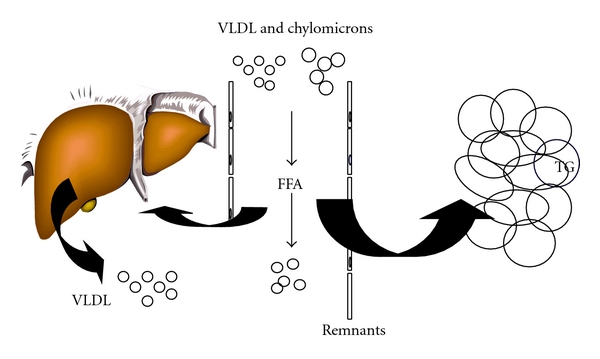
Postprandial lipid and FFA metabolism. In the postprandial phase, endogenous (VLDL) and exogenous (chylomicrons) TRL compete for clearance by LPL, which hydrolyzes TG into glycerol and FFA, leaving atherogenic remnant particles. The lipolytic rate, as well as the clearance of remnant particles by liver receptors, contributes to removal of TRL from the circulation. Adipose tissue plays a crucial role in regulating FFA concentrations by suppressing the release of FFA in the circulation and stimulating the uptake of FFA liberated from TRL by LPL. This pathway is known as the pathway of adipocyte FFA trapping.

TRLs are mainly produced in the postprandial phase. A schematic overview of postprandial TG and FFA metabolism is shown in Figure 1. Endogenous TRLs (VLDL, containing apoB100 as structural protein) and exogenous TRLs (chylomicrons, containing apoB48 as structural protein) compete for clearance by LPL, which hydrolyzes TG into glycerol and FFA, leaving atherogenic remnant particles [26]. In the postprandial phase due to limited LPL availability, competition at the level of LPL will occur resulting in accumulation of TRL. This competition is most likely when fasting hypertriglyceridemia is present. The lipolytic rate, as well as the clearance of remnant particles by liver receptors, contributes to removal of TRL from the circulation. Adipose tissue plays a crucial role in regulating FFA concentrations in the postprandial period by suppressing the release of FFA in the circulation and stimulating the uptake of FFA liberated from TRL by LPL [27]. This pathway is also known as the pathway of adipocyte FFA trapping.

**Figure 2:**
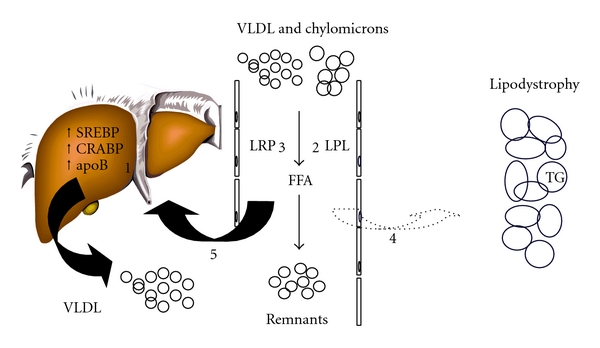
Pathogenesis of CART-related hyperlipidemia. Several factors contribute to hyperlipidemia in HIV-infected patients. PIs suppress the breakdown of the nuclear form of SREBP-1, as well as the proteasomal degradation of nascent apoB in the liver, leading to increased VLDL secretion (1). In the postprandial phase due to limited LPL availability, competition at the level of LPL will occur resulting in accumulation of TRL. This competition is most likely when fasting hypertriglyceridemia is present. In addition, impaired disposal of TRL is likely due to a defect in LPL activity (2) and delayed removal of remnant particles by liver receptors (3). Impaired FFA storage capacity (4) may lead to increased flux of circulating lipids (5) and upregulate hepatic VLDL production in patients with lipodystrophy.

A schematic overview of the proposed mechanism for hypertriglyceridemia in HIV-infected patients is shown in Figure 2. First, direct effects of PIs on hepatic TG synthesis and apoB metabolism, which have been described in detail in the previous section, may result in increased VLDL secretion. Insulin resistance and the presence of lipodystrophy may further contribute to hypertriglyceridemia. Adipocyte FFA trapping is impaired in patients with HIV-lipodystrophy, because there is not sufficient subcutaneous adipose tissue to provide the necessary buffering capacity [28]. If adipocyte FFA trapping is disturbed, then nonadipose tissues, such as the liver, skeletal muscle, and pancreas, are exposed to excessive FFA concentrations. FFA reaching the liver may contribute to an increase in VLDL synthesis [28]. In addition, impaired disposal of intestinal TRL is related to a defect in LPL activity in HIV-infected patients [28–33]. Especially the combined use of NRTIs and PIs has been associated with impaired clearance of TRL [30]. In a kinetic study, it was shown that HIV-infected patients on CART show a significant reduction in VLDL and IDL apoB fractional catabolic rates compared with HIV-negative controls, which was related to peripheral fat loss [31]. Even in fasting normolipidemic subjects, CART resulted in higher postprandial remnant lipoprotein levels, irrespective of the CART regimen [33]. Increasing evidence suggests that postprandial hyperlipidemia contributes to atherosclerosis [34–37]. Both hepatic and intestinal TRL and their remnants accumulate in the subendothelial space, where they promote atherosclerosis by the formation of foam cells [34]. Hence, current evidence suggests a proatherogenic postprandial lipoprotein phenotype in CART-treated HIV-infected patients, with accumulation of remnant lipoproteins.

## 5. Adipose Tissue

In the general population, postprandial TG and FFA metabolism is closely related to adipose tissue distribution. Especially abdominal obesity is associated with insulin resistance and hypertriglyceridemia [38]. However, absolute or partial lack of body fat may result in a similar metabolic risk profile. Several forms of congenital and acquired lipodystrophies have been related to dyslipidemia, insulin resistance, and early onset DM [39–41]. Similarly, the presence of CART-associated lipodystrophy in HIV-infected patients may play a key role in disturbed TG metabolism. 

How does the lack of body fat lead to similar manifestations to those seen with an excess of fat? There may be several explanations. First, experimental evidence indicates that subcutaneous fat may be considered a “metabolic sink” that prevents accumulation of harmful ectopic fat [39–41]. Lack of adipose tissue diverts TG and FFA to accumulate in other organs, such as the liver and skeletal muscle, leading to hepatic fat accumulation, VLDL overproduction, and insulin resistance [39–41]. In line with this hypothesis, the presence of HIV-lipoatrophy has been associated with impaired adipocyte FFA trapping [28], increased fat content in the liver [42], VLDL overproduction [24, 28], and impaired disposal of TRL [25, 29]. Most likely, the effects on dyslipidemia are resulting from lipodystrophy due to CART, although a role of HIV infection itself cannot be excluded. Second, in addition to the central role of lipid storage, adipose tissue also releases a large number of cytokines and bioactive mediators that influence body weight homeostasis, inflammation, and insulin sensitivity [43, 44]. These various protein signals are often referred to as adipocytokines and include adiponectin, leptin, IL-6, and TNF-*α*. Adiponectin levels are inversely related to indices of insulin resistance [45] and are low in patients with HIV-lipodystrophy [46, 47]. Low adiponectin levels are associated with a moderately increased CVD risk in diabetic men [48]. The differentiation of preadipocytes into mature adipocytes is a key process contributing to the normal function of adipose tissue. Subcutaneous adipocytes differ from visceral adipocytes in many respects. Compared with subcutaneous adipocytes, visceral adipocytes are hyperlipolytic and have a distinct secretion profile of adipocytokines [43, 44]. Lipodystrophy is characterized by impaired differentiation of preadipocytes to mature adipocytes [8, 9], resulting in reduced production of leptin and adiponectin [46]. Leptin deficiency and hypoadiponectinemia correlate with lipoatrophy and visceral fat accumulation in HIV-infected patients, whereas hypoadiponectinemia also appears to be associated with insulin resistance and dyslipidemia [47]. Clearly, these studies emphasize the importance of adipose tissue as an active endocrine organ involved in several metabolic and inflammatory processes that are relevant for the development of atherosclerosis. Both, excess of visceral fat and lack of subcutaneous fat, are related to impaired postprandial FFA handling, hypertriglyceridemia, and insulin resistance, which is partly mediated by adipocytokines.

## 6. Insulin Resistance

Insulin resistance is commonly seen in HIV-infected patients. Insulin resistance may result from direct effects of antiretroviral agents, effects of HIV infection, or indirect effects, such as changes in body fat distribution [49]. It has been shown that PIs induce insulin resistance in vitro by reducing insulin-mediated glucose uptake by glucose transporter 4 [50]. In HIV-negative adults, PIs reduce insulin sensitivity as early as 4 weeks after administration, without changing body fat distribution [17, 51, 52]. Direct effects of NRTIs and NNRTIs on insulin sensitivity have not been demonstrated, but these classes may contribute to insulin resistance indirectly through changes in body fat distribution. Insulin resistance has been related to excess of visceral fat, loss of subcutaneous fat, and increased waist-to-hip ratio [3–5]. Basal lipolytic rates are generally increased in patients with HIV-lipodystrophy, suggesting impaired action of hormone-sensitive lipase [53]. In addition, several studies have reported elevated FFA levels following glucose or insulin challenges, suggesting resistance to the action of insulin to suppression of lipolysis [54, 55]. The prevalence of insulin resistance among those treated with CART is up to 60 percent, depending on the criteria and techniques used [56, 57].

As a consequence of insulin resistance, abnormalities in glucose tolerance have been frequently observed in HIV-infected patients. The prevalence of diabetes mellitus (DM) was 7 percent in HIV-infected adults with lipodystrophy compared with 0.5 percent of healthy controls matched for age and BMI [58]. Impaired glucose tolerance was present in 35 percent of HIV-infected patients compared with 5 percent of matched controls [58]. The prevalence and incidence of DM have also been analyzed in the Multicenter AIDS Cohort Study [59]. In this study, 14 percent of HIV-infected men had DM compared with 5 percent of HIV-negative men (prevalence ratio of 4.4 after adjustment for age and BMI). In addition, DM was 3.1 times as likely to develop in HIV-infected men receiving CART as it was in control subjects over a three-year period of observation. Exposure to a PI-containing regimen, stavudine, or efavirenz was each independently associated with the development of DM.

## 7. Metabolic Syndrome

In the general population, several metabolic risk factors are strongly interrelated and are part of the metabolic syndrome (MS) as was elegantly described by Reaven in 1988 [60]. The MS encompasses disturbances in glucose, insulin, and lipid metabolism, associated with abdominal obesity. The National Cholesterol Education Program (NCEP) has endorsed the importance of the MS in cardiovascular risk assessment by introducing a case definition of the MS based on clinically easily obtainable anthropometric and laboratory parameters [61]. Using this definition, the MS is present when at least three out of five risk determinants (waist circumference ≥88 cm in women and ≥102 cm in men, TG ≥1.7 mmol/L, HDL-cholesterol ≤1.20 mmol/L in women or ≤1.0 mmol/L in men, glucose ≥6.1 mmol/L or blood pressure ≥130/85 mmHg) are present [61]. The MS affects 24 percent of the adult population in the U.S. [62] and 15 percent of nondiabetic adult Europeans [63] and is associated with an increased risk of CVD [64]. The MS is closely linked abdominal obesity [38], but absolute or partial lack of body fat may result in a similar metabolic risk profile [39–41]. 

Several studies focused on the prevalence and characteristics of the MS in HIV-infected patients and possible related factors. The prevalence of the MS ranges from 17 to 42 percent and is partly dependent on the definition used [65–71]. In a study of Jericó et al., the prevalence of the MS was 17 percent among HIV-infected patients and was independently associated with age, BMI, and past and present PI exposure [65]. In a report by Samaras et al., the prevalence of MS was 18 percent by NCEP criteria [66]. In this study, half of the patients had at least two features of MS but were not classified as having MS as their waist circumference was in the non-MS range. MS was more common in those currently receiving PIs and was associated with a substantially increased prevalence of DM in this specific cohort. In a study of Jacobsen et al., almost one quarter of the HIV-infected adults had the MS [67]. Most patients with the MS had low HDL-cholesterol and high TG plus ≥1 additional abnormality. The incidence of MS was higher with increasing viral load, higher BMI and higher trunk-to-limb fat ratio, and lopinavir/ritonavir or didanosine use. Mondy et al. reported that the overall prevalence of the MS was similar between HIV-infected patients (26 percent) and HIV-negative persons (27 percent), although the HIV-infected patients had a significantly smaller waist circumference, lower BMI, lower HDL-cholesterol, higher TG, and lower glucose levels, compared with the subjects from the NHANES cohort [68]. High prevalence of the MS was reported by three separate studies, ranging from 35 to 42 percent [69–71]. In the Data Collection on Adverse Events of Anti-HIV Drugs (DAD) study, for all definitions considered, there was an increasing prevalence of the MS over time, although the prevalence estimates themselves varied widely [71]. Using an NCEP definition that was modified to take account of the use of lipid-lowering and antihypertensive medication and measurement variability, an increase in prevalence from 19 percent in 2000/2001 to 42 percent in 2006/2007 was found. 

The risk of developing the MS seems to be related to HIV-infection, specific antiretroviral agents and body fat distribution [65–71]. The MS in HIV-infected patients is diagnosed mostly through low HDL-cholesterol and high TG. Frequently, HIV-infected patients do not meet waist circumference criteria for the MS, despite high rates of body fat partitioning disturbances. The high lipodystrophy prevalence rates and skewing of BMI towards normal may partly explain the relatively low prevalence of the MS in HIV-infected patients compared with that of the general population, in which higher obesity rates are found. Thus, NCEP criteria may underestimate the prevalence of the MS in HIV-infected patients. It should also be noted that the presence of the MS in HIV-infected individuals does not appear to increase CVD risk over and above that conferred by the components of the MS separately [72]. Hence, it is unclear whether the MS has additive value in cardiovascular risk assessment of HIV-infected patients.

## 8. Surrogate Markers of Atherosclerosis

Endothelial dysfunction is an early marker of atherosclerosis and can be assessed clinically by ultrasound assessment of brachial artery flow-mediated vasodilation (FMD). FMD is correlated with the severity and extent of coronary sclerosis [73] and predicts future cardiovascular events [74]. Ultrasound measurement of carotid intima-media thickness (IMT) is a well-accepted, noninvasive method of assessing early changes in vascular structure and is widely used as a surrogate marker for atherosclerotic disease [75]. Assessment of both preclinical atherosclerotic markers may provide important information on the functional and structural stages of atherosclerosis.

In a cross-sectional study of HIV-infected adults, it was shown that those on a PI-containing regimen had markedly impaired FMD compared with those not taking PIs [76]. However, FMD was not compared with an HIV-negative reference group, and the relative contributions of CART, HIV infection, and metabolic risk factors were difficult to identify. Francisci et al. performed a retrospective cohort study in HIV-infected patients before and after starting CART and matched healthy controls [77]. Soluble markers of endothelial function were significantly higher in HIV-infected patients before starting CART than in healthy controls. Short-term treatment with CART reduced some markers of endothelial dysfunction, with no differences between PIs and NNRTIs. In a prospective cross-sectional study by Ross et al*.,* endothelial markers were also higher in CART-naive patients compared with healthy controls but were similar between HIV-patients on CART and healthy controls [78]. Strong correlations were found between inflammatory cytokines and endothelial markers. Hence, both studies highlight a potential association between inflammation and endothelial activation [77, 78]. The endothelium could be activated either directly by HIV or by a leukocyte-mediated inflammatory cascade triggered by HIV infection [79–81]. In recent years, a prominent role for inflammation in the pathogenesis of atherosclerosis has emerged and circulating inflammatory molecules have been identified as markers of atherosclerotic risk [82]. Antiretroviral agents may also directly induce endothelial dysfunction. For example, when healthy volunteers were given the PI indinavir for 4 weeks, significant endothelial dysfunction was observed, independent of the lipid profile [83, 84]. Unlike the dramatic impairment seen with indinavir, the newer PIs atazanavir and lopinavir/ritonavir did not induce endothelial dysfunction in healthy subjects [85]. Finally, endothelial dysfunction has also been related to metabolic risk factors. In one study, the presence of the MS in HIV-infected patients was associated with markedly impaired FMD [69]. FMD was related to several metabolic parameters, such as dyslipidemia and insulin resistance [69].

Structural vascular abnormalities are also present in HIV-infected patients. Carotid IMT is higher in HIV-infected patients than in age-matched controls [86, 87], and progresses much more rapidly than previously reported rates in non-HIV cohorts [88]. In HIV-infected patients, IMT is related to several traditional risk factors [69, 86–88] but when a control group was added to the analysis, HIV infection was also an independent predictor of IMT [88]. Furthermore, progression of IMT has been related to low nadir CD4 cell counts [88]. Even after adjustment for traditional risk factors, HIV-infected patients have greater carotid IMT than controls [89]. In one study, the association between HIV infection and carotid IMT was similar to that of traditional risk factors, such as smoking [89]. Inflammatory and endothelial activation markers have been associated with increased carotid IMT, supporting a potential role of inflammation in CVD in HIV-infected patients [90]. Increased carotid IMT has also been related to CART. In a cross-sectional study of Jericó et al., 42 percent of the HIV-infected patients had subclinical carotid atherosclerosis, defined by carotid IMT >0.8 mm or the presence of plaque [91]. Exposure to CART was independently associated with subclinical carotid atherosclerosis in this study. Finally, HIV-infected individuals with the MS may be at increased risk for atherosclerosis based on higher carotid IMT [69, 92]. HIV-infected patients with the MS were more likely to have a carotid IMT >0.8 mm than were those without MS. Any positive coronary artery calcium score was more likely to occur for participants with MS [92]. 

**Figure 3:**
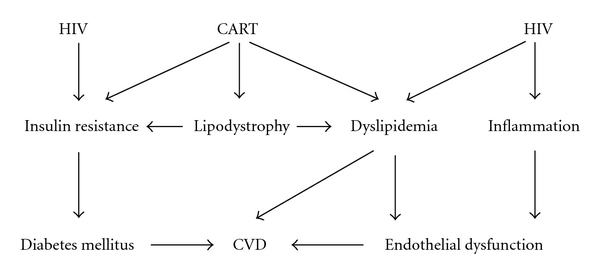
Pathogenesis of CVD in HIV-infected patients. HIV infection itself has been associated with subclinical atherosclerosis due to a low-grade inflammatory response leading to endothelial dysfunction. CART may promote atherosclerosis through its effects on body fat distribution, lipid metabolism and insulin sensitivity. Presumably, both HIV infection and CART promote atherosclerosis, either directly or indirectly via metabolic risk factors.

Taken together, most studies support the concept that HIV-infected patients are at risk for accelerated atherosclerosis. As illustrated in this section, the underlying mechanism is probably multifactorial, which is schematically depicted in Figure 3. The HIV infection itself may directly induce insulin resistance and dyslipidemia, including hypertriglyceridemia and low HDL-cholesterol. Furthermore, chronic HIV infection is associated with a proinflammatory state leading to endothelial dysfunction. CART may also promote atherosclerosis through mechanisms involving endothelial cells, either directly or indirectly via metabolic risk factors.

## 9. Cardiovascular Disease

Use of CART in HIV-infected patients has been associated with a large benefit in terms of mortality [1, 2]. In a large retrospective study, this benefit was not diminished by any increase in the rate of CVD [1]. However, this study was conducted among 36,766 patients who received care for HIV infection between 1993 and 2001, and longer-term observations and analyses are required. Since then, several studies on CVD endpoints have been published, of which most demonstrate increased CVD risk in HIV-infected patients. In the DAD study, CART was independently associated with a 26 percent relative increase in the rate of myocardial infarction (MI) per year of exposure during the first four to six years of use [6]. However, the absolute risk of MI was relatively low. Hypercholesterolemia, older age, smoking, DM, male sex, and a prior history of CVD were also associated with an increased risk of MI [6]. A central question is whether this observed risk is attributable to all classes of antiretroviral drugs or only to specific drugs. Subsequent analyses of the DAD study have demonstrated that particularly those exposed to PIs and those recently exposed to the NRTIs abacavir and didanosine had increased risk of MI [93, 94]. In contrast, no association was found between the risk of MI and exposure to NNRTIs or any of the other NRTIs [93–96]. The effect of PIs may be in part a consequence of the effects of these agents on lipid levels [93]. In contrast, associations between MI risk and abacavir and didanosine exposure were largely confined to those patients with recent exposure to the drugs and did not appear to be driven by dyslipidemia [94, 96]. Abacavir may cause vascular inflammation [96]. 

Triant et al. conducted a health care system-based cohort study using a large data registry with 3,851 HIV and 1,044,589 non-HIV patients [7]. MI rates were determined among patients receiving longitudinal care between 1996 and 2004. MI rates and cardiovascular risk factors were increased in HIV compared with non-HIV patients. The relative risk of acute MI was 1.75 in HIV-infected patients after adjustment for age, sex, race, hypertension, diabetes, and dyslipidemia. The increased MI event rate was seen over multiple age ranges and, thus, likely to be clinically significant. It should be noted that the rate of MI was higher among HIV patients in this study than in the DAD study, but this study included older patients and was from a U.S. population, with potentially different MI rates and cardiovascular risk factors than the European-based population of the DAD study. 

Inflammation appears to be an important pathogenic event in the progression of atherosclerosis [82]. Premature atherosclerosis has been reported in young adults with HIV infection in the pre-CART era [97]. Also, interruption of CART seems to be associated with an increased short-term risk of CVD [98]. Infection-induced chronic inflammation may thus contribute to the increased incidence of CVD in HIV-infected patients. In line, low CD4 cell counts have been associated with incident CVD in the HIV Outpatient Study [99]. CD4 cell count ≤500 cells/mm^3^ was an independent risk factor for incident CVD, comparable in attributable risk to several traditional CVD risk factors [99]. Thus, traditional risk factors, HIV infection, and antiretroviral agents have all been associated with CVD endpoints in HIV-infected patients.

## 10. Treatment of Risk Factors

Dyslipidemia and insulin resistance are important modifiable risk factors in HIV-infected patients. Preliminary data indicate increased cardiovascular morbidity among HIV-infected patients, suggesting that measures to reduce cardiovascular risk should be provided. It has been recommended that HIV-infected adults undergo evaluation and treatment on the basis of NCEP guidelines for dyslipidemia, with particular attention to potential drug interactions with antiretroviral agents and maintenance of virological control of HIV infection [61, 100]. In general, treatment guidelines outlined by the American Diabetes Association (ADA) and European Association for the Study of Diabetes (EASD) should be followed in HIV-infected patients with DM.

### 10.1. Lifestyle Modification

Cigarette smoking is the most important modifiable risk factor among HIV-infected patients. In the DAD study, more than 50 percent of the patients were current or former smokers, and smoking conferred a more than 2-fold risk of MI [6, 12]. Cessation of smoking is likely to reduce CVD in this population. Management of dyslipidemia must include nondrug interventions, such as a prudent diet, reduced total caloric intake, attaining ideal bodyweight, and increased physical activity. Routine aerobic activity and muscle conditioning improved trunk adiposity and lipid parameters in HIV-infected patients [101–103]. A recent randomized study showed that dietary intervention in CART-naive HIV-infected patients prevented development of dyslipidemia after 6 and 12 months [104]. Structured exercise plus diet decreased total cholesterol and TG by 11% and 21%, respectively, in HIV-infected patients [105]. HIV-infected patients with hypertriglyceridemia may also benefit from omega fatty acids [106, 107].

### 10.2. Switching Cart

Another strategy to improve dyslipidemia is switching antiretroviral agents. Switching antiretroviral agents has the potential advantage of avoiding pharmacologic intervention for elevations in lipid levels. Switching from the PI nelfinavir to the PI atazanavir reduced total cholesterol and TG with no apparent antiviral compromise [108]. Other studies have shown that switching a PI for either an NNRTI or NRTI, such as nevirapine, efavirenz, or abacavir, in patients with long-lasting viral suppression has antiviral efficacy similar to earlier PI-based combinations and may partly reverse atherogenic lipoprotein changes [109–113].

### 10.3. Lipid-Lowering Agents

Because of the potential for significant drug interactions with commonly used antiretroviral drugs, the choices of lipid-lowering agents should be limited to those agents with a low likelihood of interactions. HMG-CoA reductase inhibitors or statins are used as first-line therapy for hypercholesterolemia and reduce the risk of CVD in the general population. Several statins have been studied in HIV-infected patients. For patients receiving CART or other medications that inhibit CYP3A4, lovastatin and simvastatin should be avoided, and atorvastatin and rosuvastatin should be used with caution. In CART-treated HIV-infected patients, treatment with pravastatin was associated with improvement of the lipid profile and endothelial function [114, 115]. Others have suggested that atorvastatin and rosuvastatin are preferable to pravastatin for treatment of HIV-associated dyslipidemia, due to greater reductions in LDL-cholesterol and non-HDL-cholesterol, with similar low toxicity rates [116]. In this report, the likelihood of reaching NCEP goals for LDL-cholesterol levels was higher with the use of rosuvastatin (OR 2.1) and atorvastatin (OR 2.1) compared with that of pravastatin. A recent analysis of 829 patients has shown that dyslipidemia is more difficult to treat in HIV-infected patients than in the general population, as illustrated by smaller reductions in LDL-cholesterol and TG with lipid-lowering agents [117]. 

Fibrates, synthetic agonists for PPAR-*α*, have a well-established tolerability and efficacy profile for patients with hypertriglyceridemia and mixed hyperlipidemia. Gemfibrozil, fenofibrate, and bezafibrate have been associated with improvements of the lipid profile in HIV-infected patients [118–120]. There are no significant drug-drug interactions among PIs and fibrates. So far, the results of clinical trials on CVD endpoints with fibrates have been disappointing. However, the absolute benefits of fenofibrate are likely to be greater when MS features, including hypertriglyceridemia, are present [121]. Thus, fibrates would seem to be the preferred treatment for HIV-infected patients with dyslipidemia characterized mainly by hypertriglyceridemia. At the present time, there is no compelling reason to prefer fenofibrate to gemfibrozil in HIV-infected patients. Modest LDL-cholesterol lowering with ezetimibe has also been observed in HIV-infected patients, although its effect on CVD endpoints is unclear [122, 123].

### 10.4. Insulin-Sensitizing Agents

Because of the severity of insulin resistance in many HIV-infected patients with DM, it is reasonable to favor insulin sensitizers over insulin secretagogues. Insulin-sensitizing agents have also been studied in nondiabetic HIV-infected patients. In patients with lipoatrophy, metformin should be used with caution because further reductions in subcutaneous fat may be seen. On the other hand, studies with metformin have demonstrated significant reduction of visceral fat and improvement of insulin sensitivity, lipid levels, and endothelial function [124–127]. Thiazolidinediones, synthetic agonists for PPAR-*γ*, can be considered the preferred approach in those with lipoatrophy, given the possibility of increasing subcutaneous fat, albeit modest [127–130]. Of the thiazolidinediones, rosiglitazone improves insulin sensitivity, but most studies found detrimental effects on lipid levels [127–130]. In one study, rosiglitazone improved postprandial adipocyte FFA trapping but caused a marked increase in postprandial remnant lipoprotein levels, which may adversely affect cardiovascular risk [131]. The other registered thiazolidinedione, pioglitazone, also improves insulin sensitivity and is associated small benefits on fasting lipid profile in HIV-infected patients [132, 133]. However, pioglitazone is partly metabolized by CYP3A4, increasing the risk of clinically relevant drug interactions with PIs.

## 11. Conclusions

In HIV-infected patients, the use of CART is associated with changes in body composition, dyslipidemia, and insulin resistance. Disturbed adipose tissue distribution and altered secretion of adipocytokines may play a key role in the development of hypertriglyceridemia and insulin resistance. Presumably, both HIV infection and CART may contribute to increased CVD risk in HIV-infected patients. The absolute CVD risk, however, is still relatively small and side effects of CART should be balanced against the large benefit in terms of AIDS-related mortality. Nonetheless, as HIV-infected patients live longer on CART, CVD could become increasingly prevalent in the future. Guidelines for the evaluation and treatment of dyslipidemia have been provided. Current treatment options include lifestyle modification, switching antiretroviral agents, and use of lipid-lowering and insulin-sensitizing agents. Future research will give more insight into the pathophysiology of CVD in HIV-infected patients and the role of CART and adipose tissue.
